# Conductive Anesthesia

**Published:** 1914-11-15

**Authors:** J. E. Nyman


### CONDUCTIVE ANESTHESIA.

BY J. E. NYMAN, D.D.S.

There are two forms of toxemia apart from that of septic toxemia developing from the use of novocaine-supra-renin solutions, one of which is a systemic toxemia, which occurs almost immediately or shortly after the administration ot the local anesthetic, and the other (and it may, perhaps, at times be the graver toxemia of the two) is the purely local tissue toxemia, which is not manifested for twenty-four, thirty-six, forty-eight and sometimes seventy-two hours after the injection. This toxemia is not at all a septic toxemia, but one due to the fact that there has been a chemical degeneration of the more unstable of the two drugs used in the solution, namely, the suprarenin or adrenalin. Suprarenin bitartarie as it should be technically termed should be used, because it is more uniform and more stable, but that would undergo the typical adrenalin degeneration if brought in contact with any of the alkalies, and frequently our hypodermic syringes are boiled in solutions containing carbonate of soda. It will deteriorate sometimes because of a mere trace of alkali in distilled water, absorbed from the glass of certain bottles in which it is kept; because of the combination of heat and humidity oftentimes, or because of exposure to light.

This secondary toxemia is more or less of a gangrenous type, and may prove to be very serious. Sometimes it resembles some of the old gangrenous degenerations that we saw from injections of chloral hydrate, before that was finally abandoned. It is absolutely essential, I believe, that we use freshly distilled water, that shows no signs of any flocculent granulations in any place, and this water should at once be made into an isotonic solution, and put in a receptacle of glass which contains no alkali whatever. That solution should be made sterile by boiling for a period of anywhere from ten to twelve minutes, if necessary. I make the isotonic solution with the Ringer Beta tablets. This is the way usually that isotonic solutions are made by surgeons throughout the world.

I have in mind two cases in which colleagues consulted me because of this secondary toxemia. They were very careful about having freshly distilled water, painting the parts with iodin, and observed all of the other details of technic, and so could not account for the development of this condidition. I asked them to let me see the tablets used, and I found that they had turned a slight yellow. On questioning these men I found that these anesthetic tablets had been exposed for some time to heat, light, air and whatever humidity happened to be present, and then had been put in a white glass bottle. The tablets should be kept as nearly hermetically sealed as possible. One should never use a solution that is not absolutely colorless. The anesthetic solutions should be made not more than a half hour before you wish to use them. In a proper receptacle the solution may be kept for a week. You may determine by inspection from time to time as to whether any degeneration has set in or not, because if it has you will notice little floccular masses, and if these are present the solution should be thrown away.

As regards the relative merits of analgesia with nitrous oxid and oxygen and that of local anesthesia with suprarenin and novocain, my preference is decidedly for the latter, not only from my experience as an operator, but also as a patient under both methods. My own personal experience has been that I have been in a far more disagreeable attitude of mind and body under nitrous oxid and oxygen analgesia, although given by men of noted reputation as anesthetists in those drugs, than under the conductive anesthesia of suprarenin and novocain. Furthermore, once you have mastered the points of injection and the various methods of technic, once this careful aseptic method has become a habit with you, there is nothing the patient can do to defeat the successful establishment of anesthesia, while that is not true of nitrous oxid and oxygen analgesia in which you constantly have to have the co-operation of the patient, who may be of a nervous hysterical temperament, so that at the moment when you most need assistance it is not there. If you carry them to the point of being completely under the anesthetic, you are apt to have an anesthetic agitans develop, or an excessive flow of saliva, with increased viscosity, and the whole region made more difficult of operation.

My views are the same as those put forth by Dr. Gregg, of Pittsburg who for years was noted as one of the foremost analgesic experts in the country. After Fischer's visit to Pittsburg, Gregg said that he had used regional anesthesia about a hundred times, and nitrous oxid and oxygen but three times. There is a man who could only be convinced by the most overwhelming evidence that conductive anesthesia was superior to nitrous oxid and oxygen anesthesia.

I have heard the same thing from patients who have had both methods employed. I recall the case of a young man who presented a mouth with fourteen open cavities and four pulp extractions necessary. This young man had repeatedly had nitrous oxid and oxygen analgesia used, and he said he preferred to let the teeth go. He admitted his mouth was in a frightful state because he could not control himself—neither could any operator. It so happened that a young man about his own age, for whom I had used conductive anesthesia three times, was in my office at the time, and through him we persuaded this young man to let me try conductive anesthesia. This young man—nervous, apprehensive—would have defeated any attempt at nitrous oxid and oxygen anesthesia. The anesthesia would have had to be profound, and it is not well to have a patient in that condition while performing delicate operations on the teeth. Furthermore, nitrous oxid and oxygen analgesia renders the field of the incisors a very difficult one for operation, on account of the obstruction of the inhaler. So it has been my observation and experience— as I say, both from the standpoint of a patient and operator—that regional anesthesia, conductive anesthesia, is far more certain, definite and satisfactory to operate under, but you must observe the very strictest precautions in the preparation of the solution, both in regard to the water, the tablets and the needle and syringe and the mucous surfaces. Those things are all absolutely essential.

It is not true that validol has proved of service in cases of collapse. That of itself is of very little service. I think that most of the cases of collapse are due to the suprarenin rather than the novocain. It is the addition of the camphor —seven drops of validol camphoratum in a half ounce water—that has the good effect. It is one of the statutes of the German Empire that no one may use a local anesthetic containing either cocain, novocain, suprarenalin, or anything of that nature, without having an oil of camphor solution present to use as an antidote in case of collapse.—*Dental Review.*

